# Alterations in DNA methylation profiles in cancellous bone of postmenopausal women with osteoporosis

**DOI:** 10.1002/2211-5463.12907

**Published:** 2020-06-26

**Authors:** Yu Zhou, Ling Yang, Hong Wang, Xi Chen, Wei Jiang, Zhicong Wang, Shuping Liu, Yuehong Liu

**Affiliations:** ^1^ Department of Orthopedics People’s Hospital of Deyang City China

**Keywords:** differentially methylated genes, DNA methylation profiles, osteoporosis, postmenopausal women, signaling pathways

## Abstract

Osteoporosis is characterized by systemic microarchitecture impairment and bone loss, which ultimately lead to fragility fractures. This disease is most common in older people, especially in postmenopausal women. Cancellous bone is affected by osteoporosis earlier than cortical bone, and DNA methylation microarray analysis of the hip cancellous bone of patients with osteoarthritis revealed differential methylation. In view of the important role of cancellous bone in bone development, we examined genome‐wide DNA methylation profiles in the cancellous bone from patients with postmenopausal osteoporosis versus healthy postmenopausal women using Illumina 850K methylation microarray analysis. Under a threshold of *P* < 0.05, we obtained a total of 8973 differentially methylated genes, such as *SOX6*, *ACE*, *SYK* and *TGFB3*. Under a threshold of *P* < 0.05 and |△β| > 0.2, a total of 17 and 34 key differentially methylated genes were further identified at the promoter region and cytosine‐ phosphate‐ guanine (CpG) islands (such as *PRKCZ*, *GNA11* and *COL4A1*), respectively. *PLEKHA2*, *PLEKHB1*, *PNPLA7*, *SCD*, *MGST3* and *TSNAX* were the most common differentially methylated genes at both the promoter region and CpG islands. Five important signaling pathways, including the calcium signaling pathway, the cyclic guanosine phospho‐protein kinase G (cGMP‐PKG) signaling pathway, endocytosis, the Rap1 signaling pathway and the AMPK signaling pathway were identified. Our study may be suitable as a basis for exploring the mechanisms underlying osteoporosis in postmenopausal women.

## Graphical abstract

We studied DNA methylation profiles of cancellous bone in postmenopausal women with osteoporosis. Thirteen differentially methylated genes, including *PLEKHA2*, *PLEKHB1*, *PNPLA7*, *SCD*, *MGST3*, *TSNAX*, *PRKCZ*, *GNA11*, *COL4A1*, *SOX6*, *ACE*, *SYK* and *TGFB3*, and five related signaling pathways (calcium, cGMP‐PKG, endocytosis, Rap1 and AMPK) were identified. Our study may facilitate the identification of a novel DNA methylation molecular mechanism underlying postmenopausal osteoporosis.

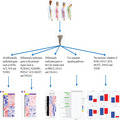

**Abbreviations**
ACE: angiotensin I–converting enzyme
Ang: angiotensin
*COL4A1*: collagen type IV alpha 1 chain
*GAN11*: G protein subunit alpha 11
*PLEKHA2*: pleckstrin homology domain containing A2
*PLEKHB1*: pleckstrin homology domain containing B1
*PNPLA7*: patatin‐like phospholipase domain containing 7
*PRKCZ*: protein kinase C zeta
*SCD*: stearoyl‐CoA desaturase
*SYK*: spleen‐associated tyrosine kinase
*TGFB3*: transforming growth factor beta 3
*TSNAX*: translin‐associated factor X

Osteoporosis, the most common bone disease, is characterized by systemic microarchitecture impairment and bone loss, which ultimately lead to fragility fractures. The bone disease is most common in older people, especially in postmenopausal women [1]. It is estimated that approximately 50% of the postmenopausal female population (>50 years old) suffer from osteoporosis [2]. The onset of postmenopausal osteoporosis is without any obvious symptoms until the fracture occurs. Generally, fragility fracture (such as in the spine, hip or femur) usually leads to pain, malformation, dysfunction and even death.

The primary reason for postmenopausal osteoporosis incidence is the disequilibrium between bone formation and bone resorption [3]. Evidence has uncovered risk factors of postmenopausal osteoporosis, such as advanced age, hypoovarianism, estrogen deficiency, increase of follicle‐stimulating hormone and luteinizing hormone, amenorrhea period, parental history of fracture, continuous calcium loss, inflammatory background and immune deficiency syndrome [2]. Serious complications and high morbidity of postmenopausal osteoporosis have attracted major focus on its pathological mechanism.

It has been found that some gene mutations, including colony‐stimulating factor 1 and low‐density lipoprotein receptor‐related protein 5, are associated with postmenopausal osteoporosis [4]. Some gene methylations are also related to postmenopausal osteoporosis. Bone DNA methylation in the sclerostin (*SOST*) promoter, bone transcripts and serum levels are associated significantly with fracture risk in postmenopausal women [5]. Methylation of bone sclerostin also impairs Sp7 transcription factor, RUNX family transcription factor 2, and estrogen receptor alpha transactivation in postmenopausal patients with osteoporosis [6]. Positive correlations between Alula hypomethylation in blood cells and several age‐related phenotypes in bone and body fat have been found in postmenopausal women with osteoporosis [7]. In addition, superoxide dismutase 1, serpin family Amember 1 and tripartite motif containing 63 could be considered as potential biomarkers for postmenopausal osteoporosis [8]. However, the pathogenesis of postmenopausal osteoporosis is still complex and not yet fully elucidated.

It has been noted that cancellous bone, composed of interwoven trabeculae, accounts for 20% of the body’s bone mass, but forms 80% of the bone surface. Mechanical properties of cancellous bone are important because they are significantly associated with fracture risk [9, 10, 11, 12, 13]. Cancellous bone is much earlier affected by osteoporosis than cortical bone at other locations of the skeleton [14]. Delgado‐Calle et al. performed DNA methylation microarray analysis on the hip cancellous bone of patients with osteoarthritis and found different methylation regions [15]. In view of the important role of cancellous bone in bone development, we tried to explore genome‐wide DNA methylation profiles in the cancellous bone from patients with postmenopausal osteoporosis. Our study may provide useful information to explore the epigenetic pathology mechanism of the disease.

## Materials and methods

### Study individuals

According to the clinical criteria of osteoporosis (*T* ≤ −2.5 standard deviations), five postmenopausal women with osteoporosis and three normal postmenopausal women (*T* ≥ −1.0 standard deviation) were recruited in this study. All of these individuals were selected outpatients from the clinic of the People’s Hospital of Deyang City. Postmenopausal women with osteoporosis and healthy postmenopausal women required natural menopause for 2–10 years. The body mass index (BMI) and age of menopause were matched between postmenopausal women with osteoporosis and healthy postmenopausal women. The inclusion criteria were as follows: none of the individuals had a history of drug use that might affect bone metabolism, such as glucocorticoids, estrogen, thyroid hormone, parathyroid hormone, fluoride, calcitonin, thiazines, barbiturates, antiepileptics, vitamin D or calcium‐containing preparations. Patients with bone metabolic diseases, such as kidney disease, liver disease, thyroid disease, diabetes, hyperprolactinemia, oophorectomy, rheumatoid arthritis, ankylosing spondylitis, absorb the adverse symptoms of chronic diarrhea, malignant tumor, blood disease, pathological fracture or traumatic fractures, hypertension, coronary atherosclerosis, myocardial infarction, cerebral infarction and infectious diseases, were excluded from this study. For genome‐wide methylation analysis, the cancellous bone of these individuals was collected for DNA extraction. Informed written consent was provided by all of the participants in this study. This study was approved by the Ethics Committee of our hospital and was performed in compliance with the Declaration of Helsinki.

### DNA isolation and bisulfite treatment

Cancellous bone tissue was first collected after removing connective tissue and adipose tissue. Then the cancellous bone tissue was quickly rinsed with 0.9% normal saline, quickly sucked off the blood with absorbent paper and cut into small pieces on ice (5 mm). Lastly, the cancellous bone tissue was put in the spiral centrifugal pipe with liquid nitrogen precooling, frozen in liquid nitrogen for more than 5 min and transferred to −80℃ refrigerator for long‐term storage. Genomic DNA was obtained from cancellous bone using the TIANamp Genomic DNA Kit (Tiangen Biotech, Beijing, China). The concentration of extracted DNA was measured using a NanoDrop 2000 spectrophotometer (NanoDrop, Thermo Scientific, Wilmington, DE, USA). Only cancellous bone samples with DNA purity from 1.8 to 2.05 were retained. Approximately 200–500 ng genomic DNA from each cancellous bone sample was chemically modified and bisulfite converted using the EZ DNA Methylation kit (Zymo Research, Irvine, CA, USA), which converts unmethylated cytosines into uracil and methylated cytosines remain unchanged during the treatment.

### Illumina 850K methylation microarray data preprocessing

To obtain the raw signal value and DetectionP of each site, we used the GenomeStudio (https://www.illumina.com/techniques/microarrays/array‐data‐analysis‐experimental‐design/genomestudio.html) software to analyze the raw data. Then quality control of the data was performed, including site control and individual control. The bisulfite conversion of genomic DNA was calculated in the process of quality control (Table S1). With that, software of lumi2.22.1 in R package [16] was used to perform the correction of fluorescence bias and quantile normalization. For probe‐type bias, the software of BMIQv1.3 (beta‐mixture quantile normalization) [17] was applied for correction of methylation level (beta value, β). Software of IMA3.1.2 in R package was utilized for analysis of differential methylation sites. In this process, the method of empirical Bayes statistics in limma [18] was put into use. Lastly, differentially methylated sites were identified under the threshold of *P* < 0.05. In addition, the cluster3.0 software was used for clustering analysis of differentially methylated sites.

### Genomic characteristic analysis of differentially methylated sites

To understand the genomic characteristic, we annotated differentially methylated sites with respect to defined CpG sites (transcription start site, 5′ UTR, 3′ UTR, body, exon, intron and intergenic region) according to the Infinium Methylation EPIC array annotation file (http://www.illumina.com).

### Functional annotation analysis of genes at differentially methylated sites

The functional annotation of identified genes at differentially methylated sites was analyzed by Kobas (http://kobas.cbi.pku.edu.cn/kobas3) [19]. Gene Ontology enrichment analysis and Kyoto Encyclopedia of Genes and Genomes signaling pathway enrichment analysis were performed. False discovery rate <0.05 was considered as significant.

### Methylation analysis at the promoter region and CpG islands

The threshold of *P* < 0.05 and |△β| > 0.2 was used to identify differentially methylated sites at the promoter region and CpG islands. Among which, △β> 0 and △β < 0 represented hypermethylation sites and hypomethylation sites, respectively. The cluster3.0 software was used for clustering analysis of differentially methylated sites in the promoter region and CpG islands.

### Electronic validation of genes in differential methylation sites

The dataset of GSE100609 (involving four cases and four normal control subjects) was used to test the expression of genes in differential methylation sites. Student’s *t* test was applied for statistical analysis. The expression result of these genes was visualized by boxplots.

## Results

### Illumina 850K methylation microarray of individuals

**Fig. 1:**
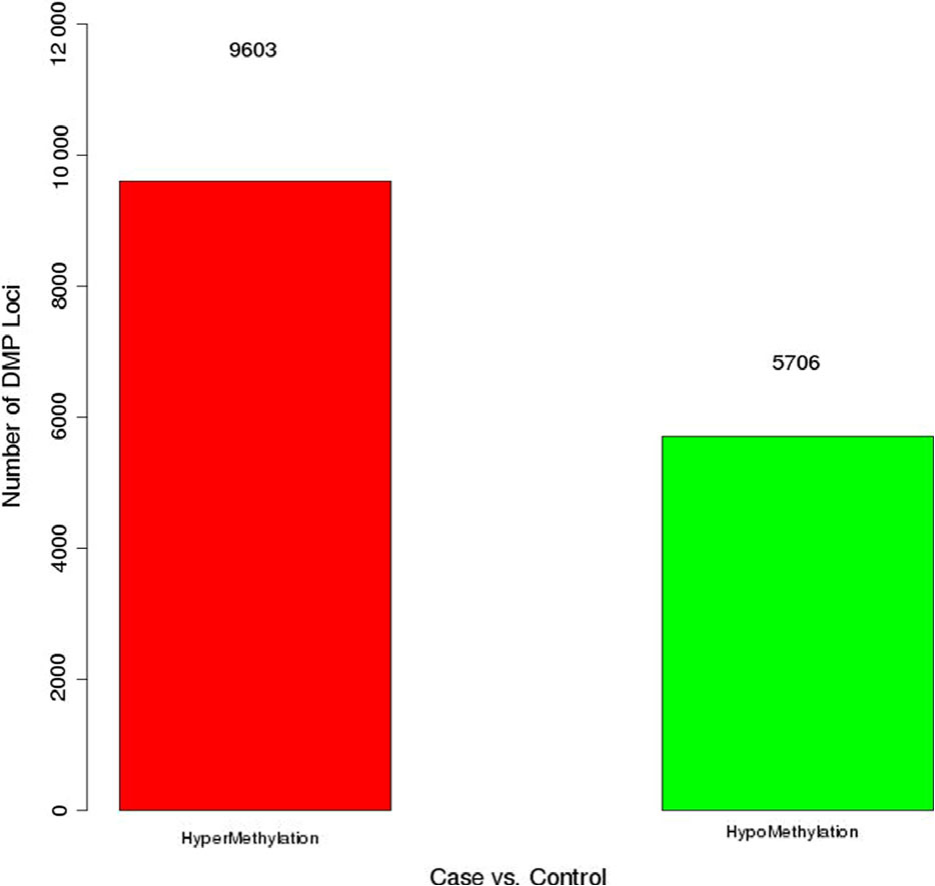
The number of differential methylation sites in postmenopausal women with osteoporosis. DMP, differentially methylated position.

**Fig. 2:**
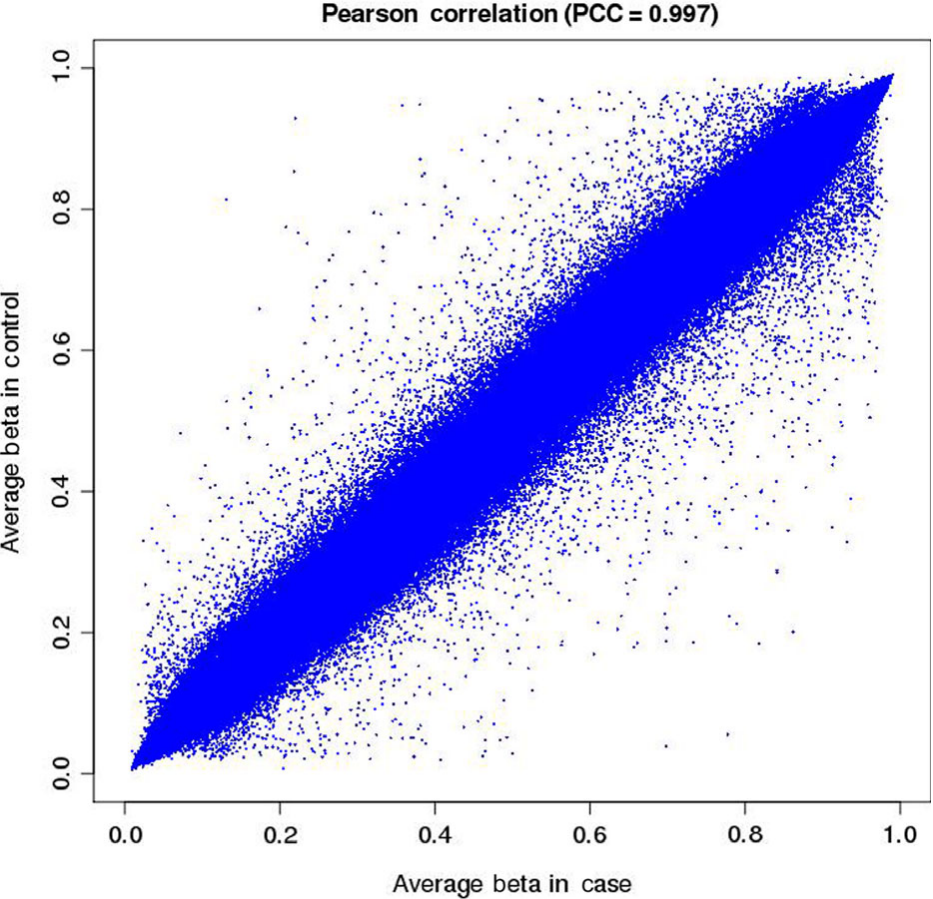
The Pearson correlation chart of each CpG site between postmenopausal women with osteoporosis and normal postmenopausal women. The *x* axis and *y* axis presented the average beta value of CpG sites in postmenopausal women with osteoporosis and normal postmenopausal women, respectively. PCC, Pearson correlation coefficient.

In this study, a total of 12 postmenopausal women with osteoporosis and 4 normal postmenopausal women were included. After a series of screenings, five postmenopausal women with osteoporosis and three normal postmenopausal women were included. The flowchart for participants selected for the study was shown in Fig. S1. In addition, the clinical information of these individuals was shown in Table 1. The DNA of cancellous bone of these individuals was used for the genome‐wide DNA methylation profiling analysis. After data processing, a total of 843 958 CpG sites were obtained. There were 9603 hypermethylation sites and 5706 hypomethylation sites in postmenopausal women with osteoporosis (Fig. 1). The Pearson correlation chart was produced for each CpG site between postmenopausal women with osteoporosis and normal postmenopausal women (Fig. 2). The Pearson correlation coefficient was 0.997, which indicated that the gene expression at the CpG sites was relatively consistent between cases and normal control subjects.

**Table 1:** The clinical information of postmenopausal women with osteoporosis and normal postmenopausal women. BMDFN, bone mineral density of femoral neck; BMDLS, bone mineral density of lumbar spine; BMDTH, bone mineral density of total hip.

Patients	Patient no.	Sample	Age (years)	BMDFN (g/cm^2^)	BMDFN (*T*‐value)	BMDLS (g/cm^2^)	BMDLS (*T*‐value)	BMDTH (g/cm^2^)	BMDTH (*T*‐value)	Weight (hg)	Height (cm)
Postmenopausal women with osteoporosis	1	Bone tissue	86	0.496	−3.6	0.607	−4.2	0.582	−3	41	140
	2	Bone tissue	80	0.616	−2.6	0.718	−3.3	0.664	−2.5	65	155
	3	Bone tissue	87	0.555	−3.1	1.053	−0.5	0.586	−3	50	155
	4	Bone tissue	89	0.529	−3.3	0.549	−4.7	0.572	−3.1	43	153
	5	Bone tissue	72	0.621	−2.6	0.82	−2.5	0.649	−2.5	64	157
Postmenopausal women	1	Bone tissue	56	0.804	−1	0.822	2.1	0.825	−1	60	150
	2	Bone tissue	78	0.748	−1	0.849	−1	0.824	−0.9	60	162
	3	Bone tissue	55	0.92	−0.1	1.346	1.9	1.138	1.3	59	155

### Identification of significantly differentially methylated sites

**Fig. 3:**
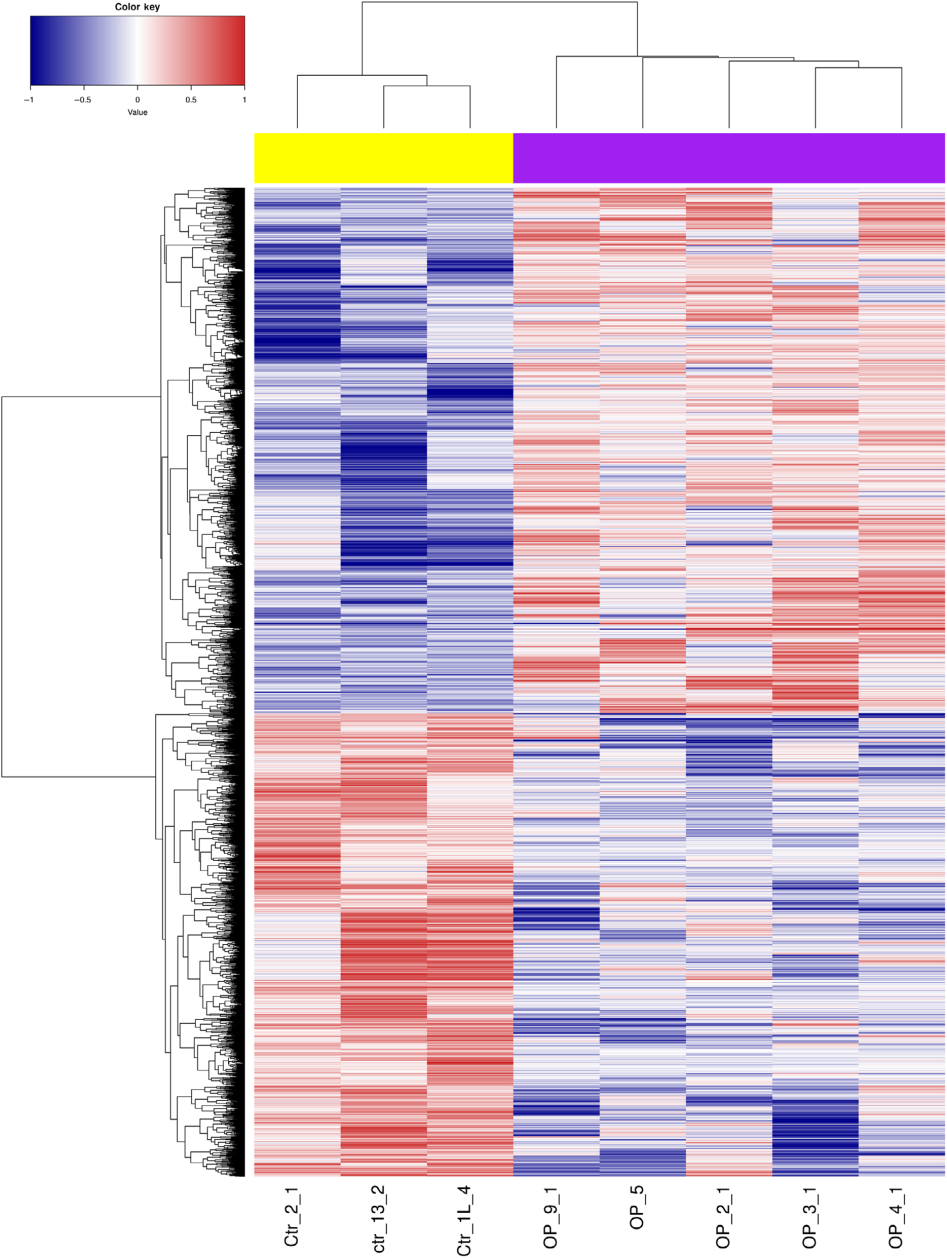
Unsupervised hierarchical clustering dendrogram of all differentially methylated sites in postmenopausal women with osteoporosis. Red, below the reference channel; blue, higher than the reference.

To analyze DNA methylation differences between postmenopausal women with osteoporosis and normal postmenopausal women, we examined the *P*‐values between both groups. A total of 15 309 CpG sites were significantly differentially methylated with *P* < 0.05, including 9603 hypermethylated sites [involving 5200 genes, such as *SOX6*, angiotensin I (Ang I)‐converting enzyme (*ACE*) and spleen‐associated tyrosine kinase (*SYK*)] and 5706 hypomethylated sites [involving 3773 genes, such as transforming growth factor beta 3 (*TGFB3*)] (Table S2). The heatmap of all significantly differentially methylated sites was shown in Fig. 3.

### Genomic features of significantly differentially methylated sites

**Fig. 4:**
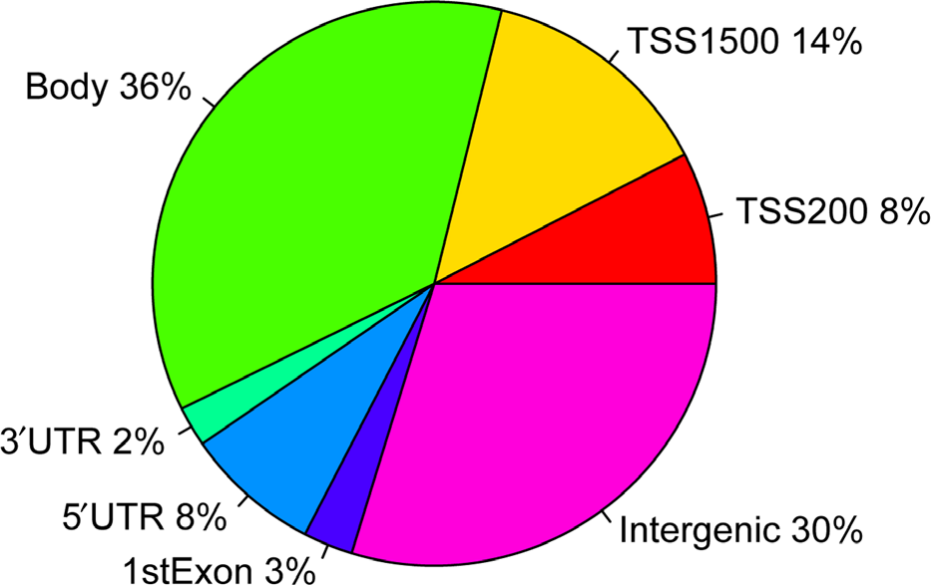
Genomic features of differentially methylated sites in postmenopausal women with osteoporosis. Graph showing percentages of differentially methylated sites according to their CpG content. TSS, transcription start site.

The CpG island of significantly differentially methylated sites was analyzed in relationship with genomic locations. Significant methylated site differences were observed between postmenopausal women with osteoporosis and normal postmenopausal women according to the CpG content. The percentage of methylation in transcription start site, 5′ UTR, 3′ UTR, body, exon, intron and intergenic region for each of the samples of postmenopausal women with osteoporosis was shown in Fig. 4. It is indicated that most of the significantly differentially methylated sites in postmenopausal women with osteoporosis were found within the body area.

### Functional enrichment analysis of genes at significantly differentially methylated sites

**Fig. 5:**
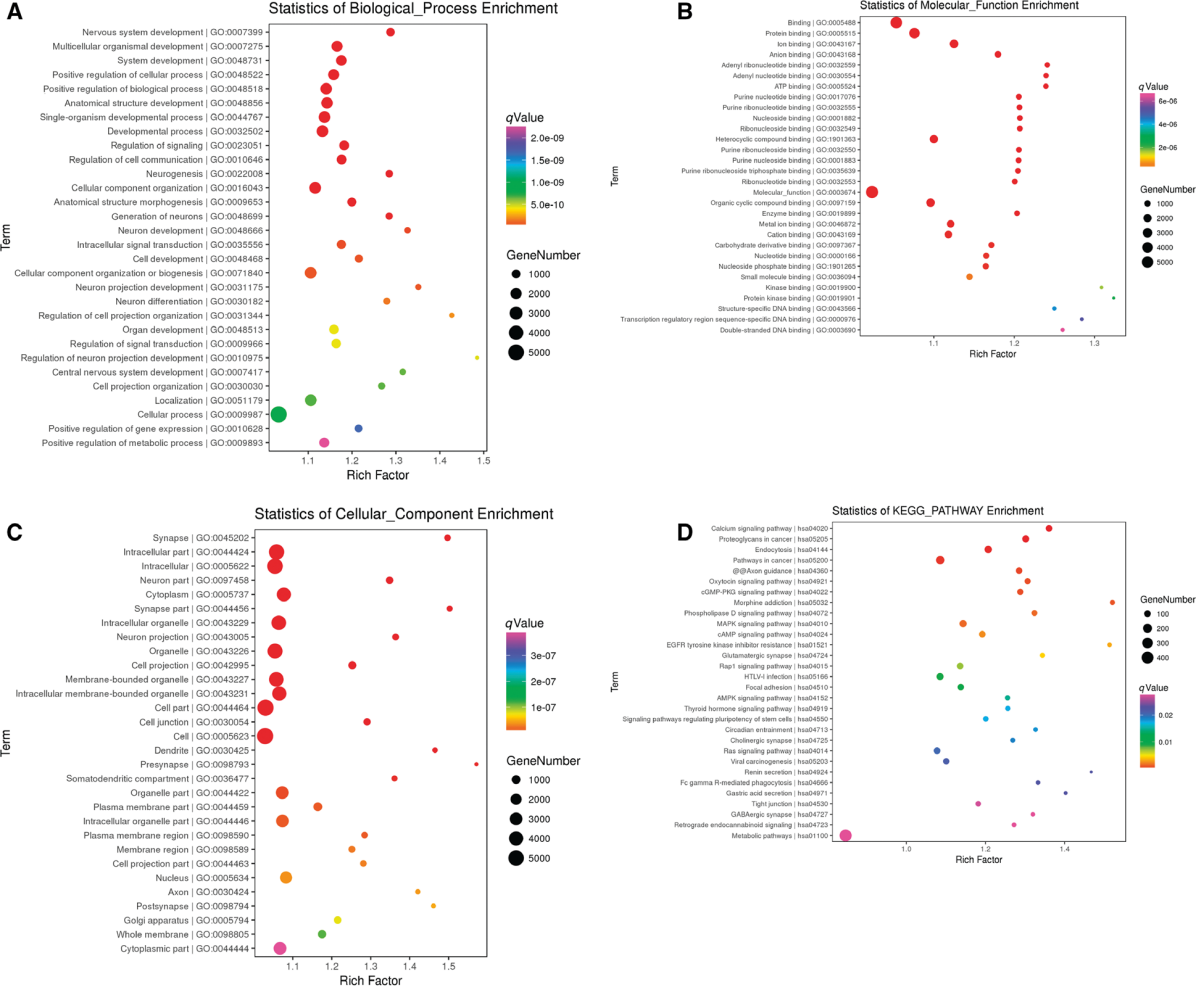
(A) Top 30 significantly enriched biological processes of genes at differentially methylated sites in postmenopausal women with osteoporosis. The *x* axis and *y* axis presented influencing factors of dot and biological processes, respectively. (B) Top 30 significantly enriched molecular functions of genes at differentially methylated sites in postmenopausal women with osteoporosis. The *x* axis and *y* axis presented influencing factors of dot and molecular functions, respectively. (C) Top 30 significantly enriched cellular components of genes at differentially methylated sites in postmenopausal women with osteoporosis. The *x* axis and *y* axis presented influencing factors of dot and cellular components, respectively. (D) Top 30 significantly enriched signaling pathways of genes at differentially methylated sites in postmenopausal women with osteoporosis. The *x* axis and *y* axis presented influencing factors of dot and signaling pathways, respectively.

To further search the molecular function of 8973 genes (5200 hypermethylated genes and 3773 hypomethylated genes) at significantly differentially methylated sites, we performed analysis of Gene Ontology and Kyoto Encyclopedia of Genes and Genomes. Functional annotation of these genes indicated that nervous system development, multicellular organismal development and system development were the most significantly enriched biological processes (Fig. 5A); binding, protein binding and ion binding were the most significantly enriched molecular functions (Fig. 5B); and synapse, intracellular part and intracellular were the most significantly enriched cellular components (Fig. 5C). In addition, the calcium signaling pathway, cGMP‐PKG signaling pathway, endocytosis, Rap1 signaling pathway and adenosine activated protein kinase (AMPK) signaling pathway were several significantly enriched signaling pathways (Fig. 5D). Significantly, several significantly differentially methylated genes were enriched in these signaling pathways. For instance, *GNA11* was involved in both the calcium signaling pathway and the cGMP‐PKG signaling pathway, *protein kinase C zeta* (*PRKCZ*) was involved in both endocytosis and the Rap1 signaling pathway, and *stearoyl‐CoA desaturase* (*SCD*) was involved in the AMPK signaling pathway. The earlier signaling pathways and enriched genes were listed in Table S3.

### Identification of significantly differentially methylated sites at the promoter region and CpG islands

**Fig. 6:**
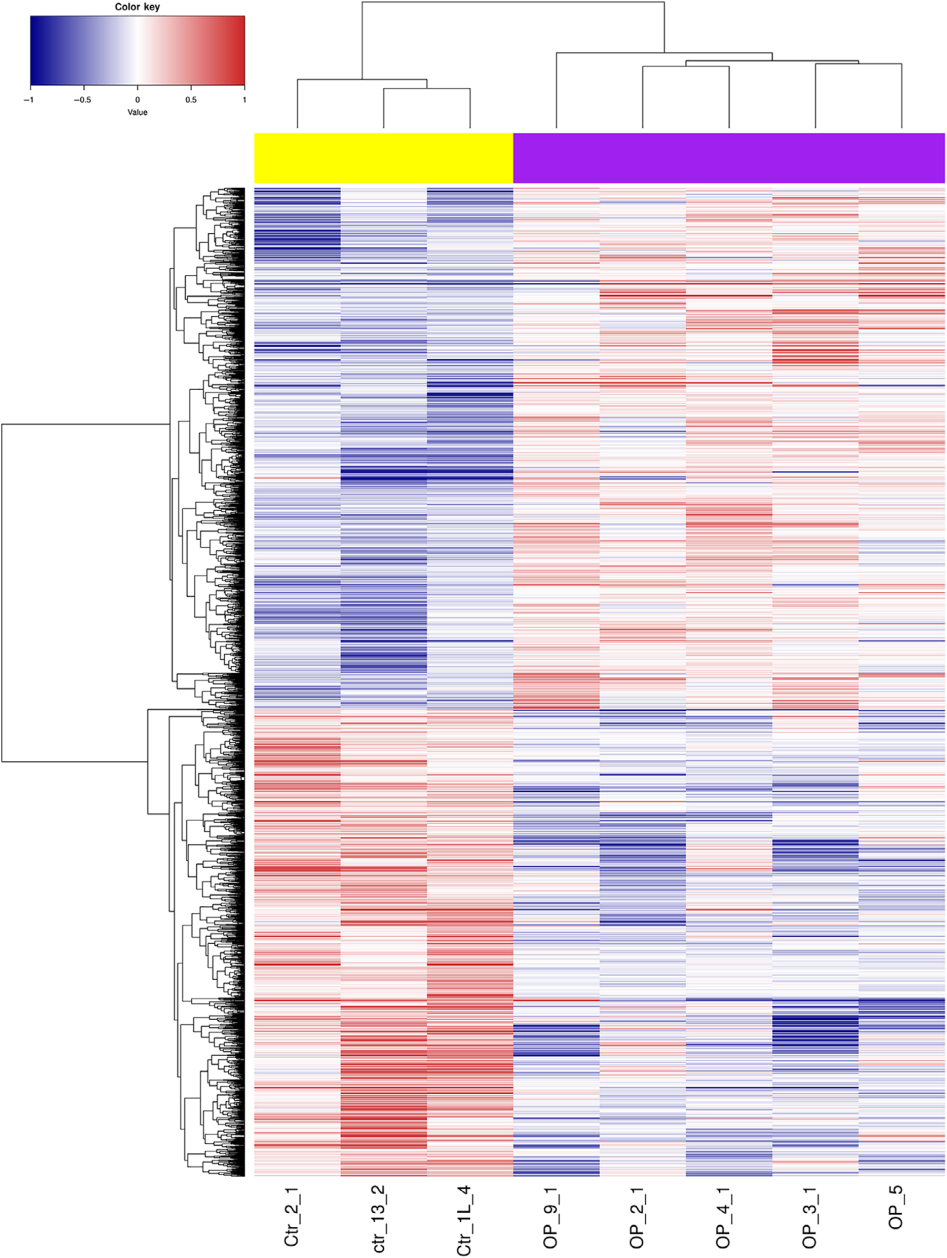
Unsupervised hierarchical clustering dendrogram of differentially methylated sites in the promoter region of postmenopausal women with osteoporosis. Red, below the reference channel; blue, higher than the reference.

**Fig. 7:**
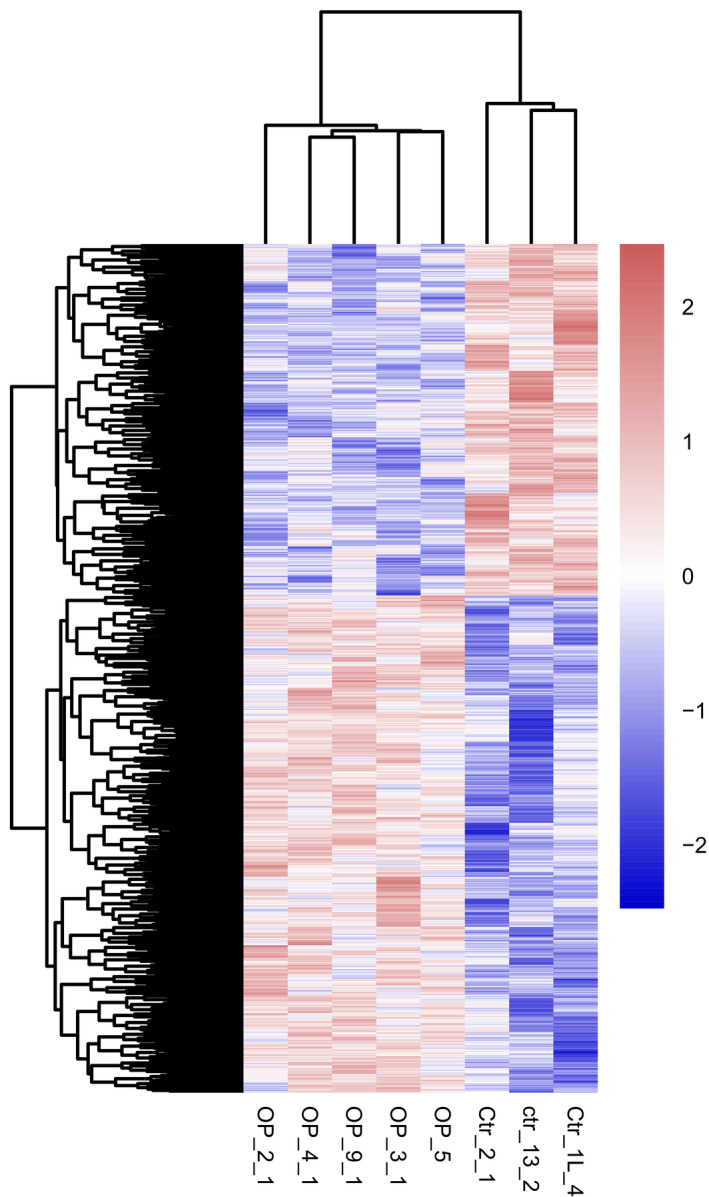
Unsupervised hierarchical clustering dendrogram of differentially methylated sites at the CpG islands in postmenopausal women with osteoporosis. Red, below the reference channel; blue, higher than the reference.

Under the threshold of *P* < 0.05, a total of 7142 and 6386 significantly differentially methylated genes were identified in the promoter region and CpG islands, respectively (data not shown). The heatmap of significantly differentially methylated genes in the promoter region and CpG islands was shown in Figs 6 and 7, respectively. In addition, under the threshold of *P* < 0.05 and |△β| > 0.2, a total of 21 (Table 2) and 63 (Table 3) key significantly differentially methylated sites were identified at the promoter region (17 differentially methylated genes involved) and CpG islands (34 differentially methylated genes involved, such as hypermethylated genes, including *GNA11* and *PRKCZ*, and hypomethylated genes, including *collagen type IV alpha 1 chain* [*COL4A1*]), respectively. It is noted that three significantly hypermethylated genes [*pleckstrin homology domain containing A2* (*PLEKHA2*), *pleckstrin homology domain containing B1* (*PLEKHB1*) and *patatin‐like phospholipase domain containing 7* (*PNPLA7*)] and three significantly hypomethylated genes (*SCD*, *MGST3* and *translin‐associated factor X* (*TSNAX*)] were the common significantly differentially methylated genes between the promoter region and CpG islands.

**Table 2:** Key differentially methylated sites at the promoter region. △β > 0: hypermethylation; △β < 0: hypomethylation.

Cg ID	*P*‐value	△β	UCSC_Refgene_Name	UCSC_CpG_Islands_Name	Relation_To_UCSC_CpG_Island
cg02121736	0.025469	0.202933	*ZNF639*	chr3:179040466‐179041632	Island
cg02564175	0.035203	0.201			
cg03422583	0.004043	0.290533	*TRABD*	chr22:50632707‐50633041	N_Shore
cg05779406	0.009116	−0.20987	*ZFAND2A*	chr7:1198965‐1200144	N_Shore
cg09019154	0.017332	−0.22153		chr8:19614131‐19615307	S_Shore
cg10117077	0.015097	0.2958	*DENND2D*		
cg10507965	0.043917	−0.30447	*SCD*	chr10:102106182‐102107722	Island
cg12201190	0.001231	0.466267	*PLEKHF1*	chr19:30164768‐30165556	N_Shore
cg13068698	0.045191	−0.20613	*DPY19L1*	chr7:35076136‐35077808	S_Shore
cg15704280	0.005264	−0.24573	*13‐Sep*	chr7:45808183‐45808745	Island
cg16848221	0.02825	−0.31247	*CLPP*	chr19:6361442‐6362002	S_Shore
cg16866567	1.38E−8	0.3714	*PLEKHA2*	chr8:38758457‐38759380	Island
cg16915828	0.007808	0.2352	*PLEKHB1*	chr11:73371800‐73372632	Island
cg17250082	0.003449	0.242067		chr6:31276241‐31276526	N_Shore
cg17873037	0.005253	−0.30533	*MGST3*	chr1:165599563‐165600574	Island
cg18559901	0.005793	0.200133	*PNPLA7*	chr9:140356314‐140356987	Island
cg19193962	0.001661	−0.21107	*TSNAX*	chr1:231663999‐231664608	Island
cg21945639	0.003314	−0.23253		chr1:200271276‐200271538	Island
cg24657788	0.005024	−0.23553	*C4orf3*	chr4:120221626‐120222007	N_Shore
cg25285484	0.007359	0.4492	*ZNF597*	chr16:3493098‐3493569	Island
cg25345738	0.040382	−0.21127	*PWP1*	chr12:108079442‐108079893	N_Shore

**Table 3:** Key significantly differentially methylated sites at the CpG islands. △β> 0: hypermethylation; △β < 0: hypomethylation.

Cg ID	*P*‐value	△β	UCSC_Refgene_Name	UCSC_CpG_Islands_Name	Relation_To_UCSC_CpG_Island
cg00545199	0.011003	0.352133	*ZFYVE28*	chr4:2305514‐2305793	
cg00947782	0.029792	0.224267	*RNF39*	chr6:30038881‐30039477	Unclassified_Cell_type_specific
cg01156747	6.41E−5	−0.58947		chr7:120519‐120758	
cg01392179	0.002224	0.315267	*FGF18*	chr5:170845760‐170848124	
cg01701555	0.027398	−0.25573		chr1:146543987‐146544439	
cg01710670	0.048918	0.2076		chr16:15018805‐15019032	
cg01818076	0.036892	0.210333		chr16:86530747‐86532994	
cg02121736	0.025469	0.202933	*ZNF639*	chr3:179040466‐179041632	Promoter_Associated
cg02148024	0.009049	0.225333	*SLC22A16*	chr6:110797297‐110798201	
cg02157463	0.028075	0.2118	*JPH3*	chr16:87648086‐87648688	
cg02599361	0.027559	0.201867	*ADAMTS2*	chr5:178547421‐178548701	
cg03019812	0.041739	0.249267	*HCG9*	chr6:29944402‐29945169	Unclassified
cg03063057	3.15E−7	0.209467	*GNA11*	chr19:3110096‐3110438	
cg03343571	0.03773	0.2408	*RNF39*	chr6:30038881‐30039477	Unclassified_Cell_type_specific
cg03929089	0.004207	−0.22047		chr4:120375746‐120376363	
cg04546413	0.040255	0.2238		chr19:29218001‐29218733	
cg05554346	0.023945	0.286467		chr4:4144575‐4145667	
cg06249604	0.04433	0.285333	*RNF39*	chr6:30038881‐30039477	Unclassified_Cell_type_specific
cg07832337	0.00619	−0.21753	*ATP2C2*	chr16:84401957‐84402497	
cg08292959	0.004096	−0.3684	*MGAT5B*	chr17:74878245‐74878455	
cg08861434	0.032836	−0.24613		chr13:112062648‐112062903	
cg08880261	0.010549	−0.47967	*UMODL1*	chr21:43547871‐43548089	
cg09194930	0.033303	−0.23093	*MT1L*	chr16:56650981‐56651384	Unclassified_Cell_type_specific
cg09279736	0.037658	0.243667	*RNF39*	chr6:30038881‐30039477	
cg10202835	0.023391	0.300867		chr5:25190503‐25191113	
cg10507965	0.043917	−0.30447	*SCD*	chr10:102106182‐102107722	Promoter_Associated
cg10930308	0.040368	0.2862	*RNF39*	chr6:30038881‐30039477	
cg11651932	0.033821	0.4722		chr8:1327331‐1327547	
cg12401798	0.034347	−0.22453	*KCNQ2*	chr20:62097193‐62098254	Unclassified_Cell_type_specific
cg12633154	0.028544	0.309	*RNF39*	chr6:30038881‐30039477	
cg12801256	0.002018	0.237533	*ST6GAL2*	chr2:107459523‐107459882	
cg13185413	0.018182	0.233667	*RNF39*	chr6:30038881‐30039477	Unclassified_Cell_type_specific
cg13401893	0.020209	0.32	*RNF39*	chr6:30038881‐30039477	
cg14188106	0.039917	0.200667	*TNXB*	chr6:32063533‐32065044	Unclassified_Cell_type_specific
cg15704280	0.005264	−0.24573	*13‐Sep*	chr7:45808183‐45808745	Promoter_Associated
cg15877520	0.048874	0.209667	*RNF39*	chr6:30038881‐30039477	Unclassified_Cell_type_specific
cg16078649	0.029298	0.280067	*RNF39*	chr6:30038881‐30039477	
cg16193862	0.000695	0.215467		chr15:24506126‐24506423	
cg16788050	0.005304	0.3398		chr11:8053245‐8053490	
cg16866567	1.38E−8	0.3714	*PLEKHA2*	chr8:38758457‐38759380	Promoter_Associated
cg16913250	1.35E−5	−0.56647	*CTTNBP2*	chr7:117512942‐117513865	
cg16915828	0.007808	0.2352	*PLEKHB1*	chr11:73371800‐73372632	Promoter_Associated
cg17550997	0.003944	0.269467		chr6:168529982‐168530307	
cg17873037	0.005253	−0.30533	*MGST3*	chr1:165599563‐165600574	Promoter_Associated
cg18025438	0.013693	0.222933		chr1:228744110‐228784168	
cg18559901	0.005793	0.200133	*PNPLA7*	chr9:140356314‐140356987	Promoter_Associated
cg18662228	0.00046	0.430533	*AGAP1*	chr2:236867652‐236867906	
cg18792536	0.002719	−0.30193	*UPK3B*	chr7:76145396‐76145781	Unclassified
cg19193962	0.001661	−0.21107	*TSNAX*	chr1:231663999‐231664608	Promoter_Associated
cg19252199	2.12E−6	−0.26647	*COL4A1*	chr13:110960924‐110961143	
cg19405842	0.003469	0.476867	*PRKCZ*	chr1:2038555‐2038863	
cg20242889	0.003057	0.246733		chr19:41317792‐41318151	
cg20249327	0.047443	0.221133	*RNF39*	chr6:30038881‐30039477	Unclassified_Cell_type_specific
cg20546215	0.024787	−0.21073		chr3:194785977‐194786549	
cg21873524	0.018211	0.355333		chr4:190942734‐190944898	
cg21945639	0.003314	−0.23253		chr1:200271276‐200271538	Promoter_Associated_Cell_type_specific
cg22172057	0.00272	0.215267	*ADARB2*	chr10:1404659‐1406219	
cg24043411	0.039615	−0.26847	*CPNE5*	chr6:36807678‐36808808	Unclassified_Cell_type_specific
cg24440302	0.047802	−0.2928	*SIGIRR*	chr11:406491‐407871	Unclassified
cg24536782	0.035538	0.260133		chr8:216352‐216828	
cg24696067	2.09E−7	0.359067	*MAD1L1*	chr7:1881181‐1881391	
cg25285484	0.007359	0.4492	*ZNF597*	chr16:3493098‐3493569	Promoter_Associated
cg26951705	0.039354	−0.44867	*ZNF787*	chr19:56612299‐56612743	
cg00545199	0.011003	0.352133	*ZFYVE28*	chr4:2305514‐2305793	
cg00947782	0.029792	0.224267	*RNF39*	chr6:30038881‐30039477	Unclassified_Cell_type_specific
cg01156747	6.41E−5	−0.58947		chr7:120519‐120758	
cg01392179	0.002224	0.315267	*FGF18*	chr5:170845760‐170848124	
cg01701555	0.027398	−0.25573		chr1:146543987‐146544439	
cg01710670	0.048918	0.2076		chr16:15018805‐15019032	
cg01818076	0.036892	0.210333		chr16:86530747‐86532994	
cg02121736	0.025469	0.202933	*ZNF639*	chr3:179040466‐179041632	Promoter_Associated
cg02148024	0.009049	0.225333	*SLC22A16*	chr6:110797297‐110798201	
cg02157463	0.028075	0.2118	*JPH3*	chr16:87648086‐87648688	
cg02599361	0.027559	0.201867	*ADAMTS2*	chr5:178547421‐178548701	
cg03019812	0.041739	0.249267	*HCG9*	chr6:29944402‐29945169	Unclassified
cg03063057	3.15E−7	0.209467	*GNA11*	chr19:3110096‐3110438	
cg03343571	0.03773	0.2408	*RNF39*	chr6:30038881‐30039477	Unclassified_Cell_type_specific
cg03929089	0.004207	−0.22047		chr4:120375746‐120376363	
cg04546413	0.040255	0.2238		chr19:29218001‐29218733	
cg05554346	0.023945	0.286467		chr4:4144575‐4145667	
cg06249604	0.04433	0.285333	*RNF39*	chr6:30038881‐30039477	Unclassified_Cell_type_specific
cg07832337	0.00619	−0.21753	*ATP2C2*	chr16:84401957‐84402497	
cg08292959	0.004096	−0.3684	*MGAT5B*	chr17:74878245‐74878455	
cg08861434	0.032836	−0.24613		chr13:112062648‐112062903	
cg08880261	0.010549	−0.47967	*UMODL1*	chr21:43547871‐43548089	
cg09194930	0.033303	−0.23093	*MT1L*	chr16:56650981‐56651384	Unclassified_Cell_type_specific
cg09279736	0.037658	0.243667	*RNF39*	chr6:30038881‐30039477	
cg10202835	0.023391	0.300867		chr5:25190503‐25191113	
cg10507965	0.043917	−0.30447	*SCD*	chr10:102106182‐102107722	Promoter_Associated
cg10930308	0.040368	0.2862	*RNF39*	chr6:30038881‐30039477	
cg11651932	0.033821	0.4722		chr8:1327331‐1327547	
cg12401798	0.034347	−0.22453	*KCNQ2*	chr20:62097193‐62098254	Unclassified_Cell_type_specific
cg12633154	0.028544	0.309	*RNF39*	chr6:30038881‐30039477	
cg12801256	0.002018	0.237533	*ST6GAL2*	chr2:107459523‐107459882	
cg13185413	0.018182	0.233667	*RNF39*	chr6:30038881‐30039477	Unclassified_Cell_type_specific
cg13401893	0.020209	0.32	*RNF39*	chr6:30038881‐30039477	
cg14188106	0.039917	0.200667	*TNXB*	chr6:32063533‐32065044	Unclassified_Cell_type_specific
cg15704280	0.005264	−0.24573	*13‐Sep*	chr7:45808183‐45808745	Promoter_Associated
cg15877520	0.048874	0.209667	*RNF39*	chr6:30038881‐30039477	Unclassified_Cell_type_specific
cg16078649	0.029298	0.280067	*RNF39*	chr6:30038881‐30039477	
cg16193862	0.000695	0.215467		chr15:24506126‐24506423	
cg16788050	0.005304	0.3398		chr11:8053245‐8053490	
cg16866567	1.38E−8	0.3714	*PLEKHA2*	chr8:38758457‐38759380	Promoter_Associated
cg16913250	1.35E−5	−0.56647	*CTTNBP2*	chr7:117512942‐117513865	
cg16915828	0.007808	0.2352	*PLEKHB1*	chr11:73371800‐73372632	Promoter_Associated
cg17550997	0.003944	0.269467		chr6:168529982‐168530307	
cg17873037	0.005253	−0.30533	*MGST3*	chr1:165599563‐165600574	Promoter_Associated
cg18025438	0.013693	0.222933		chr1:228744110‐228784168	
cg18559901	0.005793	0.200133	*PNPLA7*	chr9:140356314‐140356987	Promoter_Associated
cg18662228	0.00046	0.430533	*AGAP1*	chr2:236867652‐236867906	
cg18792536	0.002719	−0.30193	*UPK3B*	chr7:76145396‐76145781	Unclassified
cg19193962	0.001661	−0.21107	*TSNAX*	chr1:231663999‐231664608	Promoter_Associated
cg19252199	2.12E−6	−0.26647	*COL4A1*	chr13:110960924‐110961143	
cg19405842	0.003469	0.476867	*PRKCZ*	chr1:2038555‐2038863	
cg20242889	0.003057	0.246733		chr19:41317792‐41318151	
cg20249327	0.047443	0.221133	*RNF39*	chr6:30038881‐30039477	Unclassified_Cell_type_specific
cg20546215	0.024787	−0.21073		chr3:194785977‐194786549	
cg21873524	0.018211	0.355333		chr4:190942734‐190944898	
cg21945639	0.003314	−0.23253		chr1:200271276‐200271538	Promoter_Associated_Cell_type_specific
cg22172057	0.00272	0.215267	*ADARB2*	chr10:1404659‐1406219	
cg24043411	0.039615	−0.26847	*CPNE5*	chr6:36807678‐36808808	Unclassified_Cell_type_specific
cg24440302	0.047802	−0.2928	*SIGIRR*	chr11:406491‐407871	Unclassified
cg24536782	0.035538	0.260133		chr8:216352‐216828	
cg24696067	2.09E−7	0.359067	*MAD1L1*	chr7:1881181‐1881391	
cg25285484	0.007359	0.4492	*ZNF597*	chr16:3493098‐3493569	Promoter_Associated
cg26951705	0.039354	−0.44867	*ZNF787*	chr19:56612299‐56612743	
cg00545199	0.011003	0.352133	*ZFYVE28*	chr4:2305514‐2305793	
cg00947782	0.029792	0.224267	*RNF39*	chr6:30038881‐30039477	Unclassified_Cell_type_specific
cg01156747	6.41E−5	−0.58947		chr7:120519‐120758	
cg01392179	0.002224	0.315267	*FGF18*	chr5:170845760‐170848124	
cg01701555	0.027398	−0.25573		chr1:146543987‐146544439	
cg01710670	0.048918	0.2076		chr16:15018805‐15019032	
cg01818076	0.036892	0.210333		chr16:86530747‐86532994	
cg02121736	0.025469	0.202933	*ZNF639*	chr3:179040466‐179041632	Promoter_Associated
cg02148024	0.009049	0.225333	*SLC22A16*	chr6:110797297‐110798201	
cg02157463	0.028075	0.2118	*JPH3*	chr16:87648086‐87648688	
cg02599361	0.027559	0.201867	*ADAMTS2*	chr5:178547421‐178548701	
cg03019812	0.041739	0.249267	*HCG9*	chr6:29944402‐29945169	Unclassified
cg03063057	3.15E−7	0.209467	*GNA11*	chr19:3110096‐3110438	
cg03343571	0.03773	0.2408	*RNF39*	chr6:30038881‐30039477	Unclassified_Cell_type_specific
cg03929089	0.004207	−0.22047		chr4:120375746‐120376363	
cg04546413	0.040255	0.2238		chr19:29218001‐29218733	
cg05554346	0.023945	0.286467		chr4:4144575‐4145667	
cg06249604	0.04433	0.285333	*RNF39*	chr6:30038881‐30039477	Unclassified_Cell_type_specific
cg07832337	0.00619	−0.21753	*ATP2C2*	chr16:84401957‐84402497	
cg08292959	0.004096	−0.3684	*MGAT5B*	chr17:74878245‐74878455	
cg08861434	0.032836	−0.24613		chr13:112062648‐112062903	
cg08880261	0.010549	−0.47967	*UMODL1*	chr21:43547871‐43548089	
cg09194930	0.033303	−0.23093	*MT1L*	chr16:56650981‐56651384	Unclassified_Cell_type_specific
cg09279736	0.037658	0.243667	*RNF39*	chr6:30038881‐30039477	
cg10202835	0.023391	0.300867		chr5:25190503‐25191113	
cg10507965	0.043917	−0.30447	*SCD*	chr10:102106182‐102107722	Promoter_Associated
cg10930308	0.040368	0.2862	*RNF39*	chr6:30038881‐30039477	
cg11651932	0.033821	0.4722		chr8:1327331‐1327547	
cg12401798	0.034347	−0.22453	*KCNQ2*	chr20:62097193‐62098254	Unclassified_Cell_type_specific
cg12633154	0.028544	0.309	*RNF39*	chr6:30038881‐30039477	
cg12801256	0.002018	0.237533	*ST6GAL2*	chr2:107459523‐107459882	
cg13185413	0.018182	0.233667	*RNF39*	chr6:30038881‐30039477	Unclassified_Cell_type_specific
cg13401893	0.020209	0.32	*RNF39*	chr6:30038881‐30039477	
cg14188106	0.039917	0.200667	*TNXB*	chr6:32063533‐32065044	Unclassified_Cell_type_specific
cg15704280	0.005264	−0.24573	*13‐Sep*	chr7:45808183‐45808745	Promoter_Associated
cg15877520	0.048874	0.209667	*RNF39*	chr6:30038881‐30039477	Unclassified_Cell_type_specific
cg16078649	0.029298	0.280067	*RNF39*	chr6:30038881‐30039477	
cg16193862	0.000695	0.215467		chr15:24506126‐24506423	
cg16788050	0.005304	0.3398		chr11:8053245‐8053490	
cg16866567	1.38E−8	0.3714	*PLEKHA2*	chr8:38758457‐38759380	Promoter_Associated
cg16913250	1.35E−5	−0.56647	*CTTNBP2*	chr7:117512942‐117513865	
cg16915828	0.007808	0.2352	*PLEKHB1*	chr11:73371800‐73372632	Promoter_Associated
cg17550997	0.003944	0.269467		chr6:168529982‐168530307	
cg17873037	0.005253	−0.30533	*MGST3*	chr1:165599563‐165600574	Promoter_Associated
cg18025438	0.013693	0.222933		chr1:228744110‐228784168	
cg18559901	0.005793	0.200133	*PNPLA7*	chr9:140356314‐140356987	Promoter_Associated
cg18662228	0.00046	0.430533	*AGAP1*	chr2:236867652‐236867906	
cg18792536	0.002719	−0.30193	*UPK3B*	chr7:76145396‐76145781	Unclassified
cg19193962	0.001661	−0.21107	*TSNAX*	chr1:231663999‐231664608	Promoter_Associated
cg19252199	2.12E−6	−0.26647	*COL4A1*	chr13:110960924‐110961143	
cg19405842	0.003469	0.476867	*PRKCZ*	chr1:2038555‐2038863	
cg20242889	0.003057	0.246733		chr19:41317792‐41318151	
cg20249327	0.047443	0.221133	*RNF39*	chr6:30038881‐30039477	Unclassified_Cell_type_specific
cg20546215	0.024787	−0.21073		chr3:194785977‐194786549	
cg21873524	0.018211	0.355333		chr4:190942734‐190944898	
cg21945639	0.003314	−0.23253		chr1:200271276‐200271538	Promoter_Associated_Cell_type_specific
cg22172057	0.00272	0.215267	*ADARB2*	chr10:1404659‐1406219	
cg24043411	0.039615	−0.26847	*CPNE5*	chr6:36807678‐36808808	Unclassified_Cell_type_specific
cg24440302	0.047802	−0.2928	*SIGIRR*	chr11:406491‐407871	Unclassified
cg24536782	0.035538	0.260133		chr8:216352‐216828	
cg24696067	2.09E−7	0.359067	*MAD1L1*	chr7:1881181‐1881391	
cg25285484	0.007359	0.4492	*ZNF597*	chr16:3493098‐3493569	Promoter_Associated
cg26951705	0.039354	−0.44867	*ZNF787*	chr19:56612299‐56612743	
cg00545199	0.011003	0.352133	*ZFYVE28*	chr4:2305514‐2305793	
cg00947782	0.029792	0.224267	*RNF39*	chr6:30038881‐30039477	Unclassified_Cell_type_specific
cg01156747	6.41E−5	−0.58947		chr7:120519‐120758	
cg01392179	0.002224	0.315267	*FGF18*	chr5:170845760‐170848124	
cg01701555	0.027398	−0.25573		chr1:146543987‐146544439	
cg01710670	0.048918	0.2076		chr16:15018805‐15019032	
cg01818076	0.036892	0.210333		chr16:86530747‐86532994	
cg02121736	0.025469	0.202933	*ZNF639*	chr3:179040466‐179041632	Promoter_Associated
cg02148024	0.009049	0.225333	*SLC22A16*	chr6:110797297‐110798201	
cg02157463	0.028075	0.2118	*JPH3*	chr16:87648086‐87648688	
cg02599361	0.027559	0.201867	*ADAMTS2*	chr5:178547421‐178548701	
cg03019812	0.041739	0.249267	*HCG9*	chr6:29944402‐29945169	Unclassified
cg03063057	3.15E−7	0.209467	*GNA11*	chr19:3110096‐3110438	
cg03343571	0.03773	0.2408	*RNF39*	chr6:30038881‐30039477	Unclassified_Cell_type_specific
cg03929089	0.004207	−0.22047		chr4:120375746‐120376363	
cg04546413	0.040255	0.2238		chr19:29218001‐29218733	
cg05554346	0.023945	0.286467		chr4:4144575‐4145667	
cg06249604	0.04433	0.285333	*RNF39*	chr6:30038881‐30039477	Unclassified_Cell_type_specific
cg07832337	0.00619	−0.21753	*ATP2C2*	chr16:84401957‐84402497	
cg08292959	0.004096	−0.3684	*MGAT5B*	chr17:74878245‐74878455	
cg08861434	0.032836	−0.24613		chr13:112062648‐112062903	
cg08880261	0.010549	−0.47967	*UMODL1*	chr21:43547871‐43548089	
cg09194930	0.033303	−0.23093	*MT1L*	chr16:56650981‐56651384	Unclassified_Cell_type_specific
cg09279736	0.037658	0.243667	*RNF39*	chr6:30038881‐30039477	
cg10202835	0.023391	0.300867		chr5:25190503‐25191113	
cg10507965	0.043917	−0.30447	*SCD*	chr10:102106182‐102107722	Promoter_Associated
cg10930308	0.040368	0.2862	*RNF39*	chr6:30038881‐30039477	
cg11651932	0.033821	0.4722		chr8:1327331‐1327547	
cg12401798	0.034347	−0.22453	*KCNQ2*	chr20:62097193‐62098254	Unclassified_Cell_type_specific
cg12633154	0.028544	0.309	*RNF39*	chr6:30038881‐30039477	
cg12801256	0.002018	0.237533	*ST6GAL2*	chr2:107459523‐107459882	
cg13185413	0.018182	0.233667	*RNF39*	chr6:30038881‐30039477	Unclassified_Cell_type_specific
cg13401893	0.020209	0.32	*RNF39*	chr6:30038881‐30039477	
cg14188106	0.039917	0.200667	*TNXB*	chr6:32063533‐32065044	Unclassified_Cell_type_specific
cg15704280	0.005264	−0.24573	*13‐Sep*	chr7:45808183‐45808745	Promoter_Associated
cg15877520	0.048874	0.209667	*RNF39*	chr6:30038881‐30039477	Unclassified_Cell_type_specific
cg16078649	0.029298	0.280067	*RNF39*	chr6:30038881‐30039477	
cg16193862	0.000695	0.215467		chr15:24506126‐24506423	
cg16788050	0.005304	0.3398		chr11:8053245‐8053490	
cg16866567	1.38E−8	0.3714	*PLEKHA2*	chr8:38758457‐38759380	Promoter_Associated
cg16913250	1.35E−5	−0.56647	*CTTNBP2*	chr7:117512942‐117513865	
cg16915828	0.007808	0.2352	*PLEKHB1*	chr11:73371800‐73372632	Promoter_Associated
cg17550997	0.003944	0.269467		chr6:168529982‐168530307	
cg17873037	0.005253	−0.30533	*MGST3*	chr1:165599563‐165600574	Promoter_Associated
cg18025438	0.013693	0.222933		chr1:228744110‐228784168	
cg18559901	0.005793	0.200133	*PNPLA7*	chr9:140356314‐140356987	Promoter_Associated
cg18662228	0.00046	0.430533	*AGAP1*	chr2:236867652‐236867906	
cg18792536	0.002719	−0.30193	*UPK3B*	chr7:76145396‐76145781	Unclassified
cg19193962	0.001661	−0.21107	*TSNAX*	chr1:231663999‐231664608	Promoter_Associated
cg19252199	2.12E−6	−0.26647	*COL4A1*	chr13:110960924‐110961143	
cg19405842	0.003469	0.476867	*PRKCZ*	chr1:2038555‐2038863	
cg20242889	0.003057	0.246733		chr19:41317792‐41318151	
cg20249327	0.047443	0.221133	*RNF39*	chr6:30038881‐30039477	Unclassified_Cell_type_specific
cg20546215	0.024787	−0.21073		chr3:194785977‐194786549	
cg21873524	0.018211	0.355333		chr4:190942734‐190944898	
cg21945639	0.003314	−0.23253		chr1:200271276‐200271538	Promoter_Associated_Cell_type_specific
cg22172057	0.00272	0.215267	*ADARB2*	chr10:1404659‐1406219	
cg24043411	0.039615	−0.26847	*CPNE5*	chr6:36807678‐36808808	Unclassified_Cell_type_specific
cg24440302	0.047802	−0.2928	*SIGIRR*	chr11:406491‐407871	Unclassified
cg24536782	0.035538	0.260133		chr8:216352‐216828	
cg24696067	2.09E−7	0.359067	*MAD1L1*	chr7:1881181‐1881391	
cg25285484	0.007359	0.4492	*ZNF597*	chr16:3493098‐3493569	Promoter_Associated
cg26951705	0.039354	−0.44867	*ZNF787*	chr19:56612299‐56612743	

### Electronic validation of genes in significantly differential methylation sites

**Fig. 8:**
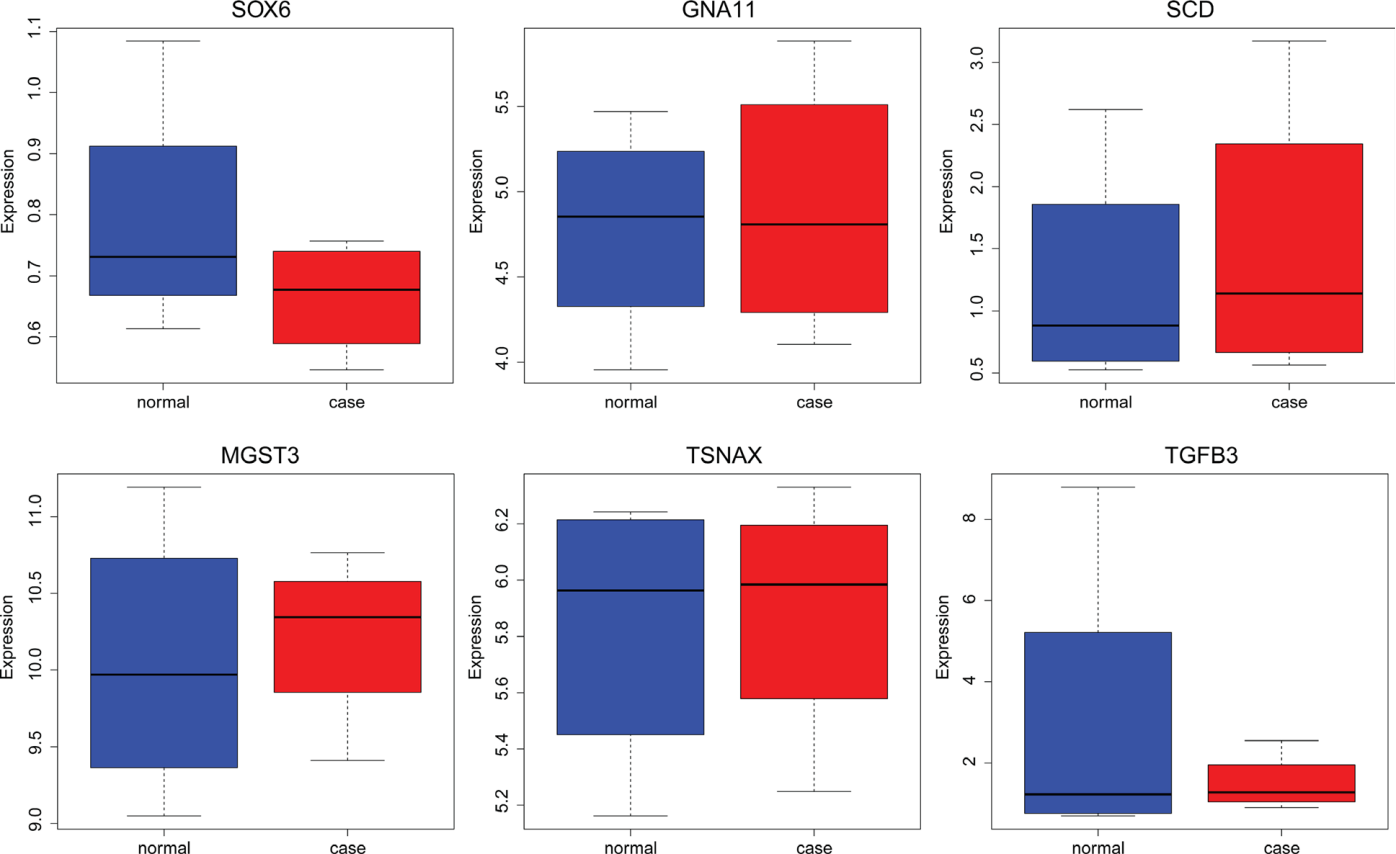
The boxplots of electronic validation of *SOX6*, *GNA11*, *SCD*, *MGST3*, *TSNAX* and *TGFB3* in the GSE100609 dataset.

In this study, two significantly hypermethylated genes (*SOX6* and *GNA11*) and four significantly hypomethylated genes (*SCD*, *MGST3*, *TSNAX* and *TGFB3*) in significantly differential methylation sites were randomly selected for validation in the GSE100609 dataset (Fig. 8). Our result showed that *SCD*, *MGST3*, *TSNAX* and *TGFB3* were up‐regulated, and *SOX6* and *GNA11* were down‐regulated with no statistical significance. The expression was consistent with the bioinformatics analysis.

## Discussion

In this study, we found that DNA methylation was involved in the process of osteoporosis in postmenopausal women. In the identification analysis of differentially methylated sites (under the threshold of *P* < 0.05 and |△β| > 0.2), three common significantly hypermethylated genes (*PLEKHA2*, *PLEKHB1* and *PNPLA7*) and three significantly hypomethylated genes (*SCD*, *MGST3* and *TSNAX*) were found between the promoter region and CpG islands. PLEKHA2 (also called TAPP2) plays an important role for phosphatidylinositol 3‐kinase‐driven cytoskeletal reorganization [20]. It is reported that PLEKHA2 is associated with juvenile idiopathic arthritis and rheumatoid arthritis [17]. The down‐regulation of PLEKHB1 was found in human osteoblast‐like cells [21]. PNPLA7 is a conserved protein in mouse and human. The expression of PNPLA7 is remarkably increased in human neonatal articular cartilage [22]. The mutation of *PNPLA7* gene (rs3812499) is related to rheumatoid arthritis [23]. It is found that SCD is up‐regulated in skeletal muscle tissues of patients with osteoporosis [24]. Moreover, high SCD activity significantly increases the risk for fracture in men [25]. Microsomal glutathione *S*‐transferase 3 (MGST3), an oxidative stress protein, is associated with rheumatoid arthritis [26]. The expression of TSNAX is found in bone marrow‐derived very small embryonic‐like cells [27]. Herein, we first found the significant expression of *PLEKHA2*, *PLEKHB1*, *PNPLA7*, *SCD*, *MGST3* and *TSNAX* in osteoporosis of postmenopausal women, which may be valuable in understanding the pathology mechanism of the disease.

Under the threshold of *P* < 0.05 and |△β| > 0.2, we also found some significantly differentially methylated genes at the CpG islands, including two significantly hypermethylated genes [*PRKCZ* and G protein subunit alpha 11 (*GAN11*)] and one significantly hypomethylated gene (*COL4A1*). It is believed that the activation of PRKCZ leads to the production of reactive oxygen species and facilitates osteoclast differentiation in synovium tissue of patients with rheumatoid arthritis [28]. In addition, PRKCZ is a potential prognosis marker for patients with osteosarcoma [29]. GAN11 is involved in skeletal growth. It is noted that GAN11 plays a key role in osteogenesis and chondrogenesis of osteoarthritis [30]. COL4A1 is significantly associated with the collarbone and thigh bone density [25]. COL4A1 is up‐regulated in bone tissue of patients with osteoporosis [31]. It is worth mentioning that regulation of the TGF‐β/Smad2/COL4A1 signaling pathway promotes osteogenic differentiation of bone marrow stromal cells and is of great significance for the new treatments strategy for postmenopausal osteoporosis [32]. Our result indicated that *PRKCZ*, *GAN11* and *COL4A1* may be associated with the development of osteoporosis in postmenopausal women.

In addition, we found four significantly differentially methylated genes, including three significantly hypermethylated genes (*SOX6*, *ACE* and *SYK*) and one significantly hypomethylated gene (*TGFB3*), under the threshold of *P* < 0.05. SRY‐box transcription factor 6 (*SOX6*), a cartilage‐expressed transcription factor, plays an important essential role in cartilage formation. The expression of *SOX6* is decreased in primary cultured osteoblasts from the patient with high bone mass [33]. In addition, the association between *SOX6* and osteoporosis has been documented [34]. By combing transcript profiling with DNA methylation analyses in bone, Reppe et al. found reduced methylation of *SOX6* in 26 osteoporotic postmenopausal women [35]. Perhaps, analytical method, sample differences or sample size could account for the inconsistent result. It is suggested that ACE could convert Ang I to Ang II in osteoblasts or osteoclasts [36]. It has been demonstrated that SYK is associated with osteoblast differentiation and osteoclasts resorption [37]. It is noted that SYK is considered as a possible target for rheumatoid arthritis treatment because of its biologic roles within bone metabolism [31]. TGFB3 could promote osteoblastogenesis at various stages. The expression of TGFB3 was detected in osteoblasts of patients with osteoporosis [38]. Our findings suggested that *SOX6*, *ACE*, *SYK* and *TGFB3* may play important roles in the bone formation of osteoporosis in postmenopausal women.

In addition to the earlier differentially methylated genes, we also found five important signaling pathways, including calcium, cGMP‐PKG, endocytosis, Rap1 and AMPK, in postmenopausal women with osteoporosis. Furthermore, several previously discussed significantly differentially methylated genes were enriched in these signaling pathways. For example, *GNA11* was involved in both the calcium signaling pathway and the cGMP‐PKG signaling pathway, *PRKCZ* was involved in both endocytosis and the Rap1 signaling pathway, and *SCD* was involved in the AMPK signaling pathway. In bone cells, osteoblasts, osteoclasts and osteocytes contain the calcium‐sensing receptor that is activated by extracellular calcium ion. Inadequate intake of calcium leads to increased bone loss. For patients with osteoporosis, ingesting adequate calcium through supplementation or diet modification is part of standard care. The phosphatidylinositol 3‐kinase/Akt/endothelial nitric oxide synthase/nitric oxide/cGMP/PKG signaling pathway is involved in the osteogenesis of bone marrow mesenchymal stem cells [39]. It is found that osteoclasts ingest bisphosphonates (a mainstay in treating and preventing osteoporosis) through endocytosis [40]. Rap1 promotes talin/integrin recognition. In osteoclasts of mice, specific deletion of Rap1 will yield similar osteopetrosis syndrome [41]. The AMPK signaling pathway is involved in osteoblastic differentiation in osteoblasts and bone cells. It is worth mentioning that the AMPK signaling pathway is associated with postmenopausal osteoporosis [42]. Our result showed that the earlier signaling pathways could be involved in the development of osteoporosis in postmenopausal women.

## Conclusions

In summary, 13 differentially methylated genes, including *PLEKHA2*, *PLEKHB1*, *PNPLA7*, *SCD*, *MGST3*, *TSNAX*, *PRKCZ*, *GNA11*, *COL4A1*, *SOX6*, *ACE*, *SYK* and *TGFB3*, and 5 related signaling pathways (calcium, cGMP‐PKG, endocytosis, Rap1 and AMPK) were identified in postmenopausal women with osteoporosis in this study. Our study may provide a novel DNA methylation molecular mechanism of postmenopausal osteoporosis. However, there are limitations of our study. First, the sample size was small. A large number of subjects is needed for further research. Second, some *in vitro* experiments, such as fluorescence quantitative PCR, western blotting and immunohistochemistry, are further needed to validate the expression of identified differentially methylated genes in large numbers of cancellous bone samples. Third, we did not investigate the deeper molecular mechanism of the disease. Additional cell experiments and animal models are further needed for investigation.

## Author contributions

HW, WJ, ZW and SL analyzed and interpreted the data. YZ and LY were major contributors in writing the manuscript. YL and XC designed the project. All authors read and approved the final manuscript.

## Conflict of interest

The authors declare no conflict of interest.

## Supporting information

Fig. S1. The flow charts for participants selected for the study.

Table S1. The bisulfite conversion of genomic DNA in the process of quality control.

Table S2. 15309 CpG significantly differentially methylated sites with P < 0.05.

Table S3. Enriched signaling pathways of significantly differentially methylated genes.
