# Supplementary figures and images for: Alterations in DNA methylation profiles in cancellous bone of postmenopausal women with osteoporosis
Source: FEBS Open Bio. 2020 Jun 26;10(8):1516–31. doi: 10.1002/2211-5463.12907 (PMC7396431; doi:10.1002/2211-5463.12907)

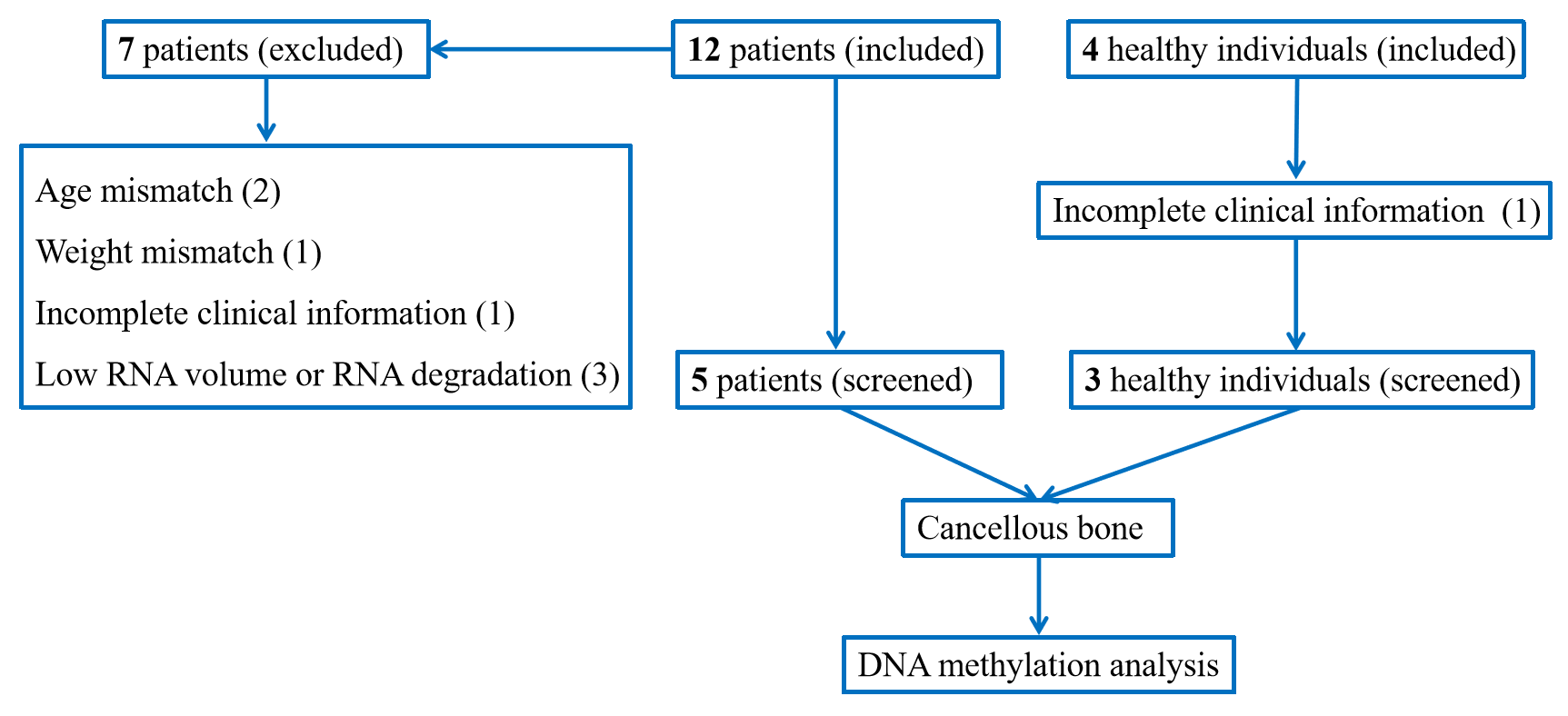

Supplement: Supplementary file 1 — Fig. S1. The flow charts for participants selected for the study. [file FEB4-10-1516-s001.tif]
