# Supplementary material for: Alterations in DNA methylation profiles in cancellous bone of postmenopausal women with osteoporosis
Source: FEBS Open Bio. 2020 Jun 26;10(8):1516–31. doi: 10.1002/2211-5463.12907 (PMC7396431; doi:10.1002/2211-5463.12907)
Supplement: Supplementary file 2 — Table S1. The bisulfite conversion of genomic DNA in the process of quality control. [file FEB4-10-1516-s002.doc]

**Supplementary Table 1 The bisulfite conversion of genomic DNA in the process of quality control**

| **Sample_ID** | **Detected_CpG** | **All_CpG** | **Conversion rate** |
| --- | --- | --- | --- |
| Patient | 865081 | 865918 | 0.999033396 |
|  |  |  |  |
| Patient | 864960 | 865918 | 0.99889366 |
|  |  |  |  |
| Patient | 865123 | 865918 | 0.999081899 |
|  |  |  |  |
| Patient | 865207 | 865918 | 0.999178906 |
|  |  |  |  |
| Patient | 865102 | 865918 | 0.999057647 |
|  |  |  |  |
| Normal controls | 864983 | 865918 | 0.998920221 |
|  |  |  |  |
| Normal controls | 865076 | 865918 | 0.999027622 |
|  |  |  |  |
| Normal controls | 865024 | 865918 | 0.99896757 |
